# Chemical and Electrochemical Synthesis of Platinum Black

**DOI:** 10.1038/s41598-017-01040-8

**Published:** 2017-04-21

**Authors:** S. E. Stanca, F. Hänschke, A. Ihring, G. Zieger, J. Dellith, E. Kessler, H.-G. Meyer

**Affiliations:** Leibniz Institute of Photonic Technology, Albert-Einstein-Straße 9, 07745 Jena, Germany

## Abstract

We present electrochemical and chemical synthesis of platinum black at room temperature in aqueous and non-aqueous media. X-ray analysis established the purity and crystalline nature. The electron micrographs indicate that the nanostructures consist of platinum crystals that interconnect to form porous assemblies. Additionally, the electron micrographs of the platinum black thin layer, which was electrochemically deposited on different metallic and semiconductive substrates (aluminium, platinum, silver, gold, tin-cooper alloy, indium-tin-oxide, stainless steel, and copper), indicate that the substrate influences its porous features but not its absorbance characteristics. The platinum black exhibited a broad absorbance and low reflectance in the ultraviolet, visible, and infrared regions. These characteristics make this material suitable for use as a high-temperature resistant absorber layer for the fabrication of microelectronics.

## Introduction

The unique properties of platinum allow for several exacting applications^1, 2^. In addition to its uses as a standard in metrology and photometry^3, 4^ and an efficient catalyst^5–7^ or electrocatalyst^8^ in the chemical industry, stable platinum is widely applied to the fabrication of microelectronics^9, 10^. Since the last century, much scientific effort has focused on producing novel platinum structures with new optical values^11–14^ while preserving its electrical and thermal conductivity. In this context, we report the synthesis of platinum as black entities that are characterized by a broad absorbance and low reflectance not only in the visible region but also in the infrared region from 500 cm^−1^ (20 µm) to 50000 cm^−1^ (0.2 µm). The known method for platinum black powder manufacturing consists of heating at 500 °C in molten sodium nitrate for 30 minutes with ammonium chloroplatinate. Next, the molten mass is poured into water followed by boiling, washing, and reducing the resulting platinum dioxide with gaseous hydrogen to platinum black^15^. In contrast to this laborious pathway, we prepare platinum black using a one-step method that is analogous to the gold salt reduction method^16^ in water and isopropanol, respectively. The synthesis of the desired platinum “black” occurs by reaction of platinum salt (PtCl_4_ 5H_2_O) with sodium tetrahydroborate (NaBH_4_) in a precise ratio at room temperature (§Methods). The obtained platinum black possesses a high purity and can be further utilized for thin layer preparation. However, in microelectronic fabrication, a highly localized presence of platinum black is highly desirable. This objective can be achieved using an electrochemical method rather than a chemical approach. Kohlrausch pioneered the electrochemical preparation of platinum black on platinum electrodes in 1897^17^ starting from an electrolyte composition consisting of platinum chloride:lead acetate:water (1:0.008:30) at a current density of 0.03 A/cm^2^. The electroplating bath^18^, which utilizes various platinum complexes, such as [PtCl_6_]^2−^, [Pt(NH_3_)_4_]^2+^, [Pt(NO_2_)_4_]^2−^, affords glossy platinum not the black one. The electrode modification with platinum black was obtained in recent decades by Ilic *et al*.^19^ via a metal vapour deposition approach in the electric arc, which is similar to that reported by Pfund for metallic ‘‘blacks’’^20^ in 1933. Recently, in 2013, Chekin *et al*.^21^ produced a grapheme oxide-platinum black nanocomposite, which exhibits electrocatalytic activity. The potential for a highly localized platinum black deposition has been demonstrated with the Kohlrausch method. Therefore, we adapted this method to achieve nanolayers of platinum black on different cathodic materials including platinum, gold, silver, stainless steel, copper, indium-tin oxide (ITO) and aluminium (§Methods).

## Results

**Figure 1:**
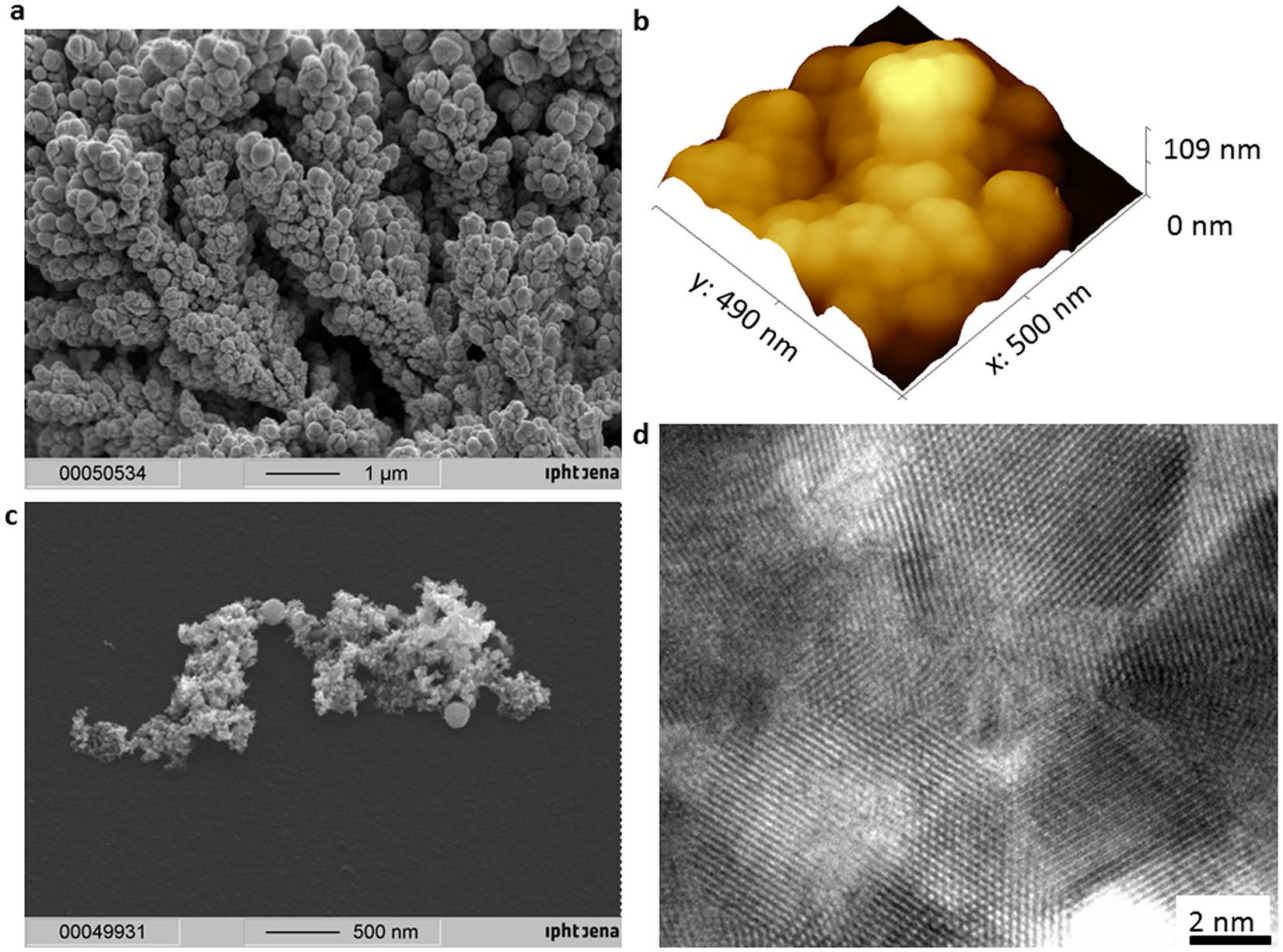
Platinum black morphology. (**a**) SEM image of the platinum black electrochemically synthesized on platinum substrate. (**b**) AFM image of the platinum black; (**c**) SEM image of the platinum black immobilized on carbon substrate. (**d**) HRTEM image of the platinum crystals.

**Figure 2:**
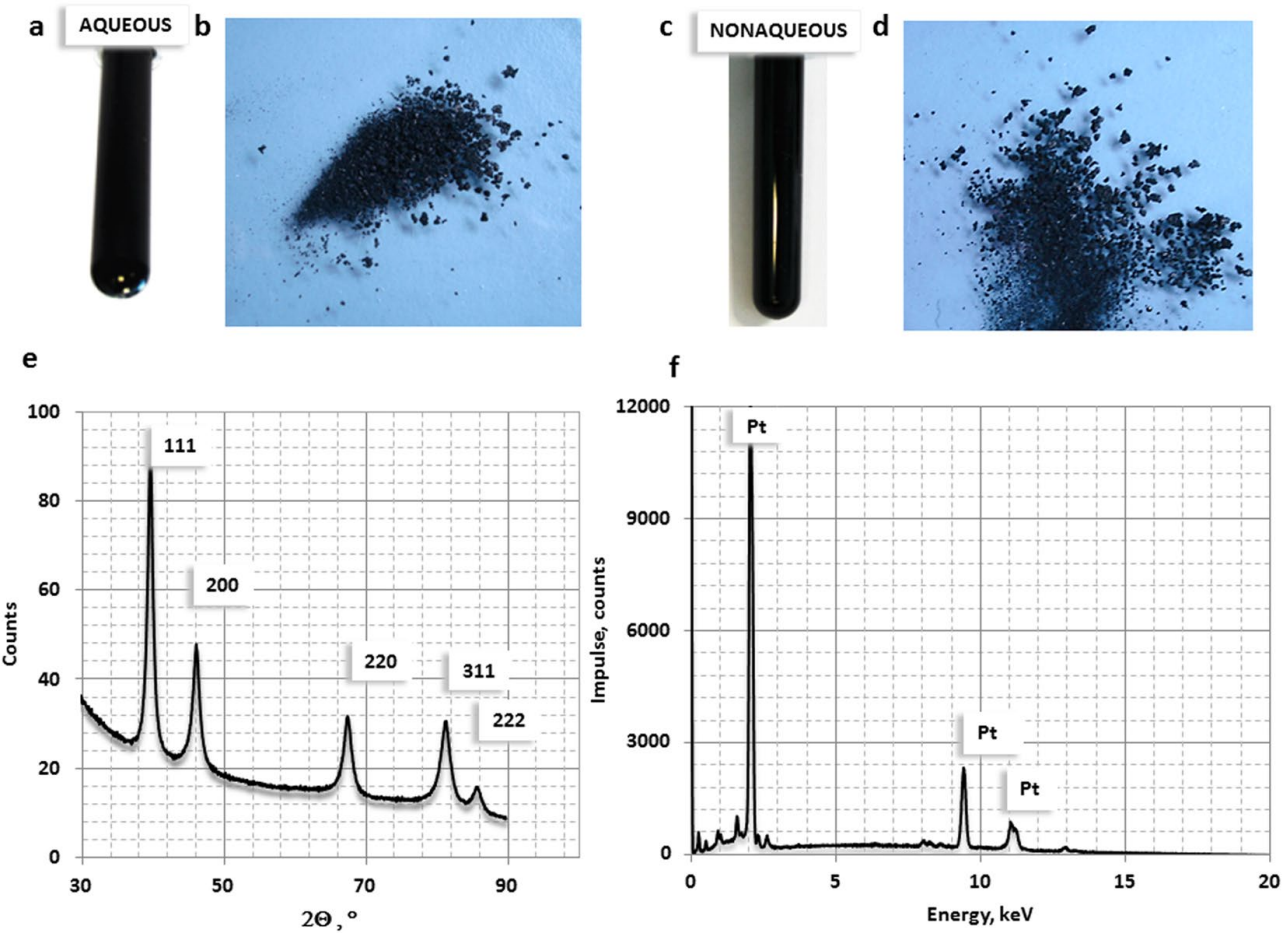
Platinum black structure from X-ray analysis. (**a**,**b**) Photograph of the colloidal platinum and dried platinum crystals synthesized in aqueous medium; (**c**,**d**) Photograph of the colloidal platinum and dried platinum crystals synthesized in non-aqueous medium. (**e**) XRD pattern; (**f**) EDX pattern.

The as-prepared platinum was investigated using scanning and transmission electron microscopy (SEM and TEM), atomic force microscopy (AFM), (Fig. 1) energy dispersive X-ray spectrometry (EDX) and X-ray diffraction (XRD) (Fig. 2). The electron micrographs suggest that the nanostructures consist of platinum crystals that interconnect to form porous assemblies, which ensures an increased surface area. The EDX results confirmed the purity, and the XRD results confirmed the crystalline nature of the platinum black. The very low IR transparency makes it a valuable candidate for a stable absorber in infrared microsensors.

### Morphology of platinum black

SEM and AFM images of the platinum reveal a nanostructured network (Fig. 1a–c). The crystallinity of the platinum network was confirmed by the mean of HRTEM (Fig. 1d).

#### Structural characterization of the platinum black

X-ray diffraction (XRD) and Energy dispersive X-ray spectrometry (EDX) indicate the structure and the composition of platinum black (Fig. 2). Within the instrument detection limit, the XRD pattern of the platinum identifies the peaks of faced centred cubic (fcc) polycrystalline platinum with a small texture leading to an increase of the 111 reflex (Fig. 2e). By using the Scherrer formula and evaluating the spectrum shown in Fig. 2e we have estimated the crystallite size to be approximately 10 nm. This result is an average value over all spatial directions. Because of the high background in the spectra the contribution of the smallest dimension (i.e. largest FWHM) may show a higher measuring error. The crystal size of approximate 10 nm is confirmed by scanning electron microscopy operated in the backscattered mode. For energy dispersive X-ray (EDX) measurements micrometer sized structures were deposited on amorphous carbon substrates. The spectra recorded at E_0_ = 20 keV clearly show the lines related to platinum (Fig. 2f).

#### Light absorption characteristics

**Figure 3:**
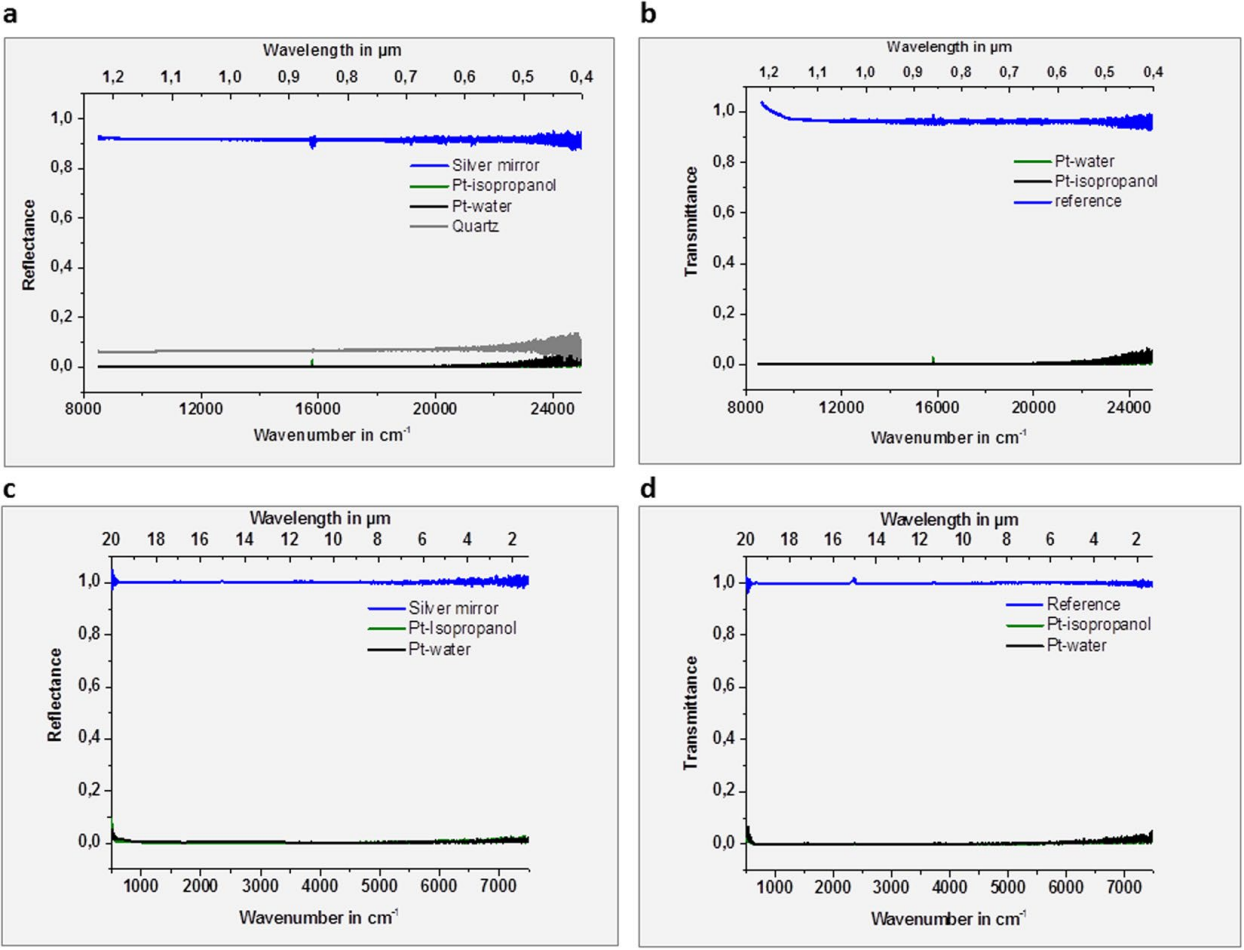
Light transmission characteristics. (**a**,**b**) Reflectance and transmittance Vis-NIR spectra of the platinum powder synthesized in water (**a**) and in isopropanol (**b**), respectively; the powder was immobilized on quartz slides; (**c**,**d**) Reflectance and transmittance FTIR spectra of the platinum powder synthesized in water (**a**) and in isopropanol (**b**), respectively. Reference reflectance: silver mirror; reference transmittance: air (blue line).

The platinum black absorbance spectra in the visible (Vis) and infrared (IR) are presented in Fig. 3. The Vis-NIR spectra recorded from 25000 cm^−1^ to 8000 cm^−1^ (corresponding to 0,4 to 1,25 µm wavelength) and the IR spectra recorded from 8000 to 500 cm^−1^ (corresponding to 1.25 to 20 µm wavelength) indicate low reflectance and transmittance, respectively a high absorbance in the whole region of investigation (Fig. 3a–d). Due to above presented broad absorbance and low reflectance in ultraviolet, visible, infrared domain, from 500 cm^−1^ (20 µm) to 50000 cm^−1^ (0.2 µm), platinum black can be applied as a high-temperature resistant absorber layer in the fabrication of microelectronics.

## Discussions

### Synthesis of platinum black (aqueous and non-aqueous media)

This study provides evidence for the one-step synthesis of platinum black. Most likely, the *in situ* generated hydrogen molecules:

1 $${{\rm{NaBH}}}_{4}+4{{\rm{H}}}_{2}{\rm{O}}\to 4{{\rm{H}}}_{2}+{\rm{NaB}}{({\rm{OH}})}_{4}$$

reduce the platinum ions to neutral platinum.

2 $${{\rm{PtCl}}}_{4}+2{{\rm{H}}}_{2}\to {\rm{Pt}}+4{\rm{HCl}}$$

The resulting nanostructures exhibit a micrometre size “oakum” like aspect. 10 nm crystals are associated in porous assemblies, as indicated by electron microscopy. The size of these assemblies may be controlled by the reductive agent to precursor ratio and/or the reaction temperature while maintaining the black aspect of the colloid. The size of the crystals can also be controlled by initialization of the platinum seed at the temperature of ice followed by a slow heating growth procedure. The platinum black is recovered from the colloid by centrifugation and can be further utilized as an absorber layer. The unique optical properties of platinum black allows for its application in various areas of research and industry.

### Electrochemical formation of platinum black as a thin layer on different materials in aqueous and non-aqueous (isopronanol) media

The electrochemical method from Kohlrausch was adapted to prepare a thin layer of platinum black on different metallic and semiconductive substrates, such as aluminium, platinum, silver, gold, tin-cooper alloy, indium-tin-oxide, stainless steel, and copper. The anodic material (i.e., platinum wire) ensures stability against oxidation. The oxygen formation can occur at the anode as follows:

3 $$4{{\rm{HO}}}^{-}\to {{\rm{O}}}_{2}+4{{\rm{e}}}^{-}+2{{\rm{H}}}_{2}{\rm{O}}$$

The chloride ions, which can also be oxidized to chloride gas, are suddenly precipitated with the lead ions, leaving the electrolyte:

4 $$2{{\rm{Cl}}}^{-}+{{\rm{Pb}}}^{2+}\to {{\rm{PbCl}}}_{2}\downarrow $$

At the cathode, the reduction of platinum ions occurs:

5 $${{\rm{Pt}}}^{4+}+4{{\rm{e}}}^{-}\to {\rm{Pt}}\downarrow $$

along with gaseous hydrogen formation:

6 $$2{{\rm{H}}}^{+}+2{{\rm{e}}}^{-}\to {{\rm{H}}}_{2}\uparrow $$

A cathodic secondary reaction involving the reduction of the carbon ion

7 $${{\rm{C}}}^{3+}+2{{\rm{e}}}^{-}\to {{\rm{C}}}^{1+}$$

from acetate to acetic aldehyde cannot be excluded:

8 $${{\rm{CH}}}_{3}{{\rm{COO}}}^{-}+2{{\rm{e}}}^{-}+3{{\rm{H}}}^{+}\to {{\rm{CH}}}_{3}{\rm{COH}}+{{\rm{H}}}_{2}{\rm{O}}$$

The mechanism for the formation of atomic platinum at the cathode remains unclear. The literature does not mention the existence of reaction (5). Therefore, we assume that in the first sub-reaction (9), Pt^2+^ is formed, which is then reduced by hydrogen as follows:

9 $${{\rm{Pt}}}^{4+}+2{{\rm{e}}}^{-}\to {{\rm{Pt}}}^{2+}$$

10 $${{\rm{Pt}}}^{2+}+2{{\rm{e}}}^{-}+{{\rm{H}}}_{2}\to {\rm{Pt}}\downarrow +2{{\rm{H}}}^{+}$$

**Figure 4:**
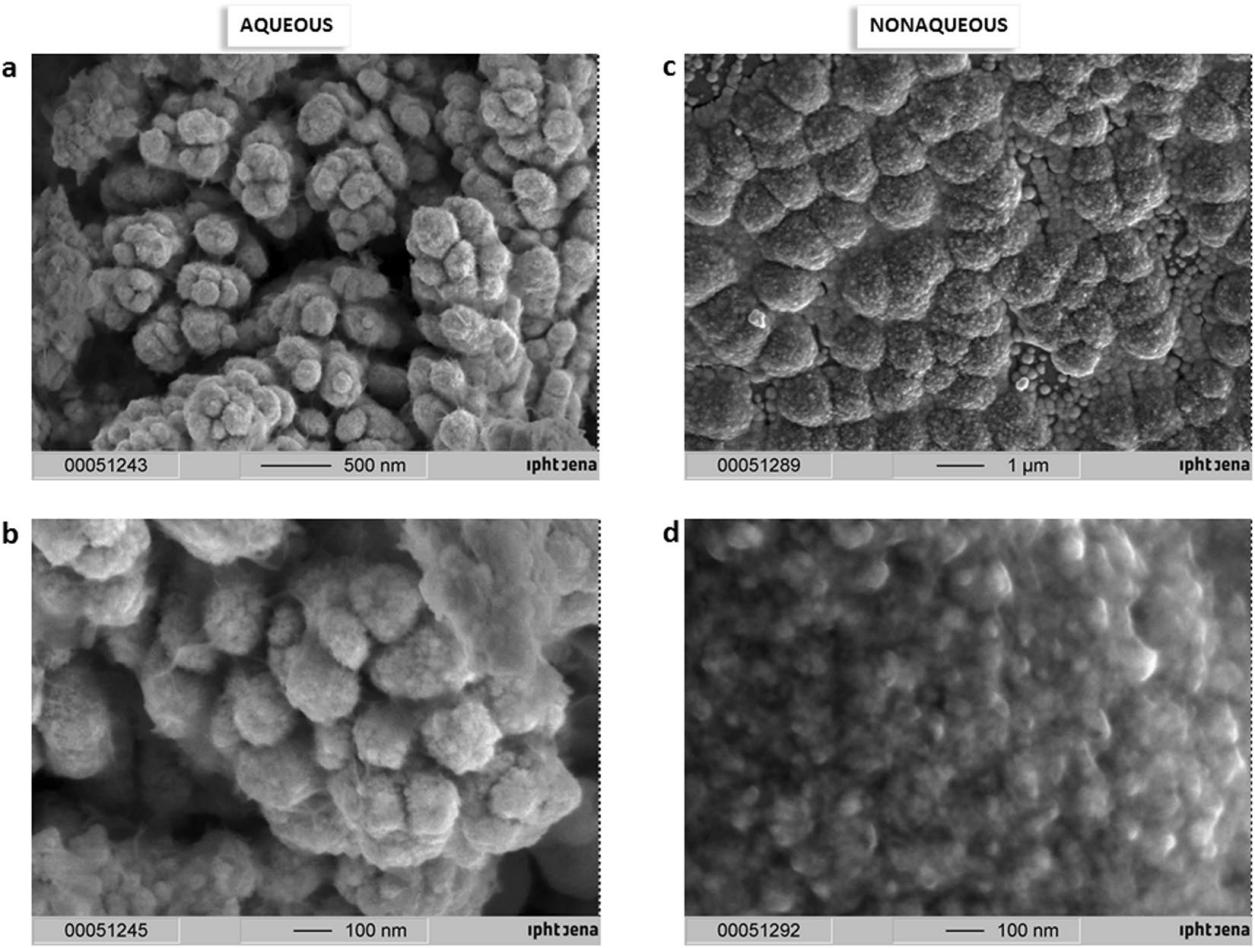
SEM of electrosynthesized platinum black layer on copper: (**a**) in aqueous, (**b**) in non-aqueous (isopropanol) media.

**Figure 5:**
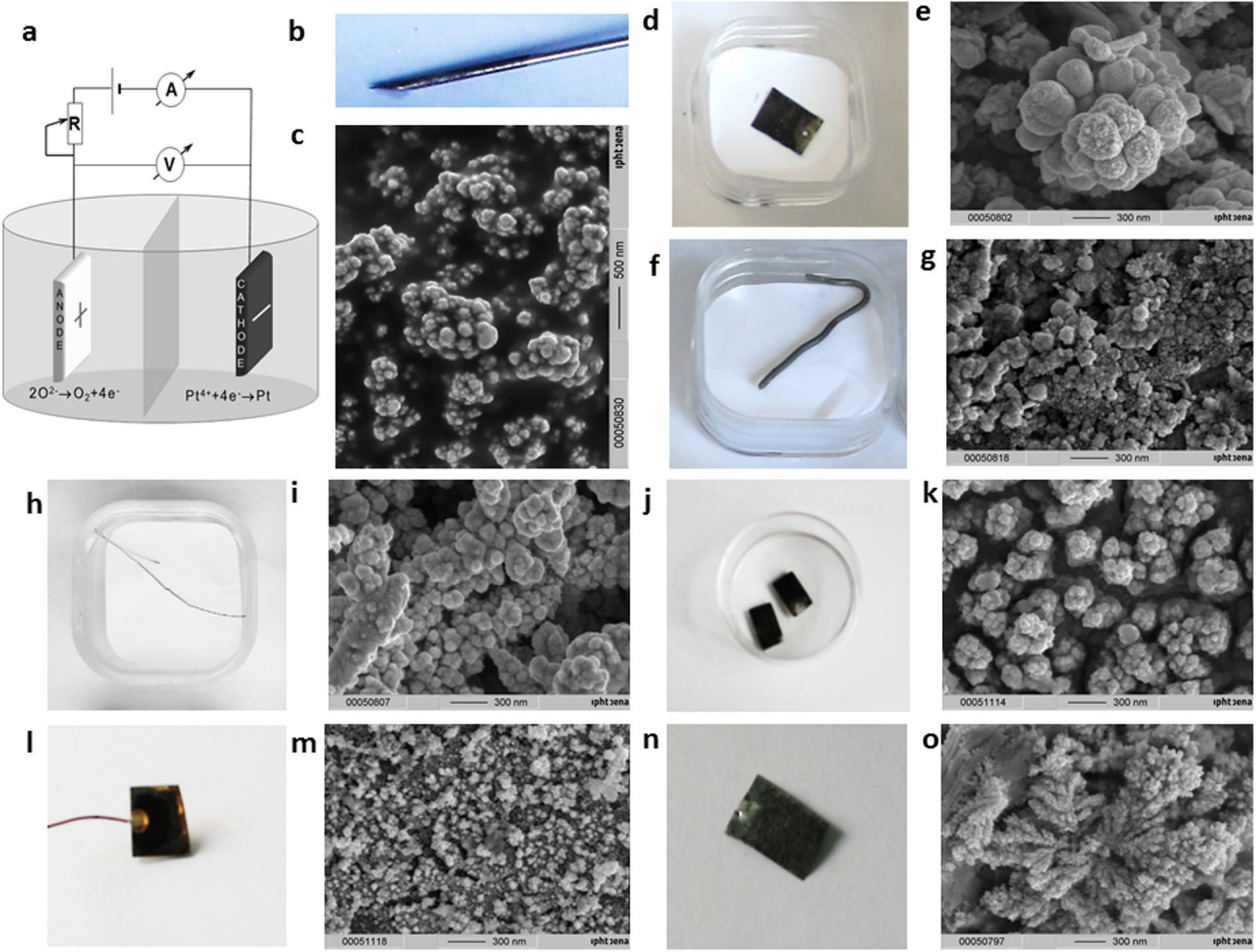
Electrochemically immobilized platinum black layer on different substrates. (**a**) The schematic representation of the electrochemical setup; (**b**) Photograph of the Pt on stainless steel substrate; (**c**) SEM image of the Pt on stainless steel indicated in (**b**); (**d**) Photograph of the Pt on Pt substrate; (**e**) SEM image of the Pt on Pt plate indicated in (**d**); (**f**) Photograph of the Pt on Ag substrate; (**g**) SEM image of the Pt on Ag indicated in (**f**); (**h**) Photograph of the Pt on Sn-Cu alloy substrate; (**i**) SEM image of the Pt on Sn-Cu alloy indicated in (**h**); (**j**) Photograph of the Pt on ITO substrate; (**k**) SEM image of the Pt on ITO indicated in (**j**); (**l**) Photograph of the Pt on Au substrate; (**m**) SEM image of the Pt on Au indicated in (**l**); (**n**) Photograph of the Pt on Al substrate; (**o**) SEM image of the Pt on Al indicated in (**n**).

**Figure 6:**
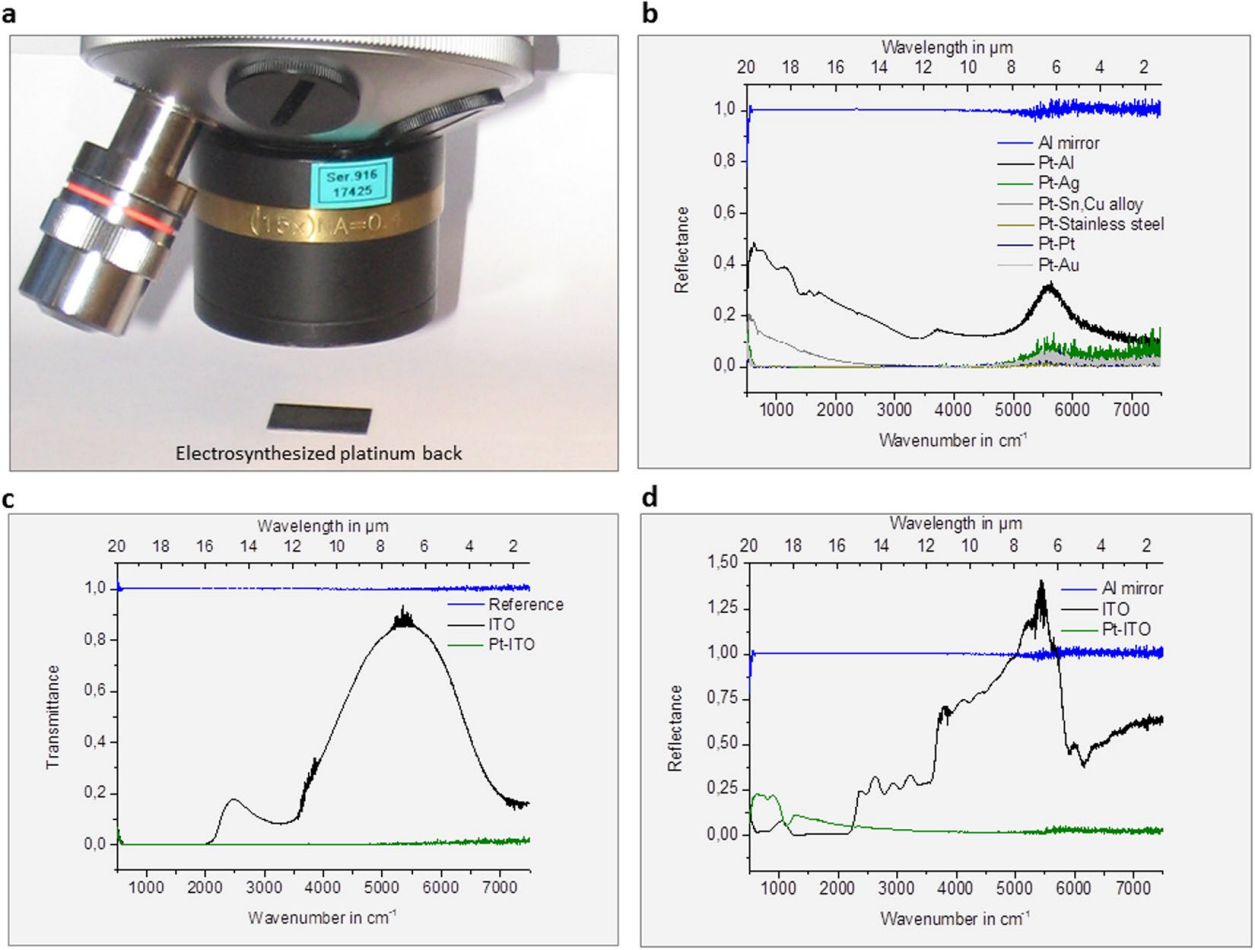
FTIR spectra of the electrosynthesized platinum black layer. (**a**) The photograph of one of the setup-section; (**b**) FTIR reflectance spectra of platinum black on Pt, Al, stainless steel, Sn, Cu alloy, Ag, and Au. (**c**,**d**) Transmittance and reflectance FTIR spectra of the platinum electrochemically deposited on Indium-Tin Oxide (ITO) substrate. Reflectance reference: aluminum mirror; transmitance reference: air.

To the best of our knowledge, for the first time, the synthesis of a platinum black layer on a copper cathode in a non-aqueous medium (isopropanol) under an electrical current of 0.1 A/cm^2^ in 180 s (Fig. 4) was successfully carried out. The electrodeposition of black platinum was analysed using EDX, and the results confirmed the presence of platinum on the electrode. The SEM images and photos of the platinum black structures obtained in both non-aqueous and aqueous media on the previously mentioned modified cathodes are shown in Figs 4 and 5. A cauliflower-like structure was observed in all the studied cases. Although all the substrates led to porous black platinum, the SEM images indicate that the substrate influences its final form. This porous structure allows for broad absorption over a broad electromagnetic region (UV-Vis and IR). Additionally, the FTIR spectra of the platinum black layers indicate low reflectance for all substrates (Fig. 6). A slightly higher reflectance was observed for the aluminium substrate due to the shorter electrolysis time. In these experiments, we demonstrated the electrochemical preparation of platinum black as a broad optical absorber in aqueous and non-aqueous media on different conductive substrates. This method can be applied for highly localized deposition of platinum black in the construction of microelectronics. In addition, we have described the chemical synthesis of platinum black in aqueous and non-aqueous media. The synthesis began with an impure platinum salt, which yields 57.5% platinum chloride, and based on X-ray analysis, we obtained highly pure platinum black crystals. The broad absorbance and low reflectance of the platinum black in the wavelength region from 0.2 µm to 20 µm allows for its application as a high-temperature resistant optical absorber layer for the fabrication of microelectronics. Due to a broad resonance in the visible region, this material has the potential for application to the construction of sensitive solar cells or as a substrate for Raman and IR spectroscopy. The high specific surface area of platinum black could prove valuable for applications in catalysis.

## Methods

### The preparation of platinum black

0.1 g platinum salt (Platinum IV chloride: PtCl_4_ 5H_2_O, MW 336.90 g·mol^−1^ Merck KGaA S5505147/003/8.07347.0005) dissolved in 10 mL water, separately 0.2 g of reduction agent (Sodium tetrahydroborate NaBH_4_, MW 37.83 g·mol^−1^ Roth, Art. No. 4051.1) dissolved in 2 mL water at 18–20 °C. NaBH_4_ solution is added in drops to the platinum salt solution. The platinum salt is reduced by hydrogen, which is slowly released from NaBH_4_ in the solution. The dispersion is homogenized by this hydrogen convection and the external stirring is not required. The appearance of the black colour indicates the completion of the synthesis (Fig. 2a), the colloidal state is assured by NaCl which is formed as additional product during the reaction and act as an efficient surfactant in this case. After approximate 3 hours of settling, the black precipitate is recovered from colloid (i.e. centrifugation, filtration or sedimentation with NaCl solution) and washed three times with water. The synthesis was repeated in isopropanol (Fig. 2c) instead of water following the same proportions. The product is black in both cases. Html color codes (http://html-color-codes.info/) indicates #000000 of black platinum synthesized in water and #1C1C1C in isopropanol (Fig. 2a–d).

### Electrochemical synthesis of platinum black on different cathodic materials

The setup consist of a classical electrolysis cell with two electrodes. Pt wire serves as anode. As cathode has been successively used: Pt plate, aluminum foil, silver wire, Sn, Cu alloy wire, Au layer, ITO glass, copper wires. To synthesize platinum black in aqueous media, we adapted a method from Kohlrausch: 0.1 g PtCl_4_ and 0.002 g Pb(CH_3_COO)_2_ in 10 mL water was used as an electrolytic bath. Under an electrical current density of 0.03–0.09 A/cm^2^, the time of electrolysis varies from one material to the other between 30 s and 120 s. To synthesize platinum black in isopropanol media, 0.1 g PtCl_4_ and 0.002 g Pb(CH_3_COO)_2_ in 10 mL isopropanol was used as an electrolytic bath. The electrical current density was 0.1 A/cm^2^ and the electrolysis time 180 s.

### Scanning Electron Microscopy (SEM)

SEM measurements were performed with a field emission microscope JSM-6300F (JEOL, Tokyo, Japan). The energy of the exciting electrons was mostly 5 keV. Beside the detector for secondary electrons (SEI), Everhart-Thornley type, the system is equipped with different detector types (semiconductor and YAG type) for backscattered electrons. The sample preparation was done by the deposition of 10 µL droplets of the particle dispersion on polished amorphous carbon substrates (sigradur) and dried in air. Because of the low atomic number of carbon this strategy helps to increase the imaging contrast.

### Energy dispersive X-ray spectrometry (EDX)

All energy dispersive X-ray analyses were done using a state of the art 30 mm² silicon drift detector (SDD) by BRUKER (BRUKER Nano GmbH, Berlin, Germany) and the Esprit spectra evaluation software package. The specified energy resolution of the detector at 5.9 keV (Mn-Kα) amounts 129 eV.

### Atomic force Microscopy (AFM)

Scanning force microscopy were carried out using a Dimension edge AFM system (BRUKER Nano GmbH, Karlsruhe, Germany). The system was operated in tapping mode using edged silicon probes (TESP; BRUKER Nano). The specified tip radius is less than 10 nm.

### Transmission Electron Microscopy (TEM)

5 µL of the particle dispersion, were deposited on a carbon coated 400 mesh copper grid. After 1 min of adsorption the excess liquid was blotted off with filter paper. Dried samples were examined by the mean of High Resolution Transmission Electron Microscopy (HRTEM) using a TEM JEOL JEM-3010 operating at 300 KeV.

### X-Ray diffraction

The X-ray diffraction analysis has been performed with an X’pert Pro Instrument (PANanalytical, Almelo, Netherlands) using Cu-Kα_1,2_ radiation. The Scherrer equation was used for the determination of the crystallite sizes.

### Visible, Near Infrared and Infrared Spectroscopy

The visible, near infrared and infrared spectra of the platinum specimens were measured with the Attenuated Total Reflectance (ATR) Mode and the Transmission Mode in a FTIR-Spectrometer (Bruker Instrument), respectively. The spectra were recorded with a resolution of 1 cm^−1^ in the spectral range 25000–500 cm^−1^.
